# Interactions between cannabis use and chronic pain on sleep architecture: Findings from in-home EEG recordings

**DOI:** 10.1016/j.neurot.2025.e00785

**Published:** 2025-11-12

**Authors:** Tracy W. Brown, Francesca M. Filbey

**Affiliations:** School of Behavioral Brain Sciences, Department of Psychology, University of Texas at Dallas, Richardson, TX, United States

**Keywords:** N3, Slow-wave sleep, Pain, Cannabis, EEG

## Abstract

Pain and sleep disturbances are primary reasons for medicinal cannabis use. Cannabis influences both pain and sleep through its modulation of the endocannabinoid system, which regulates pain and sleep signaling. Despite their interconnected roles, the effects of cannabis and chronic pain on sleep architecture have been studied mainly in isolation. An integrated understanding is needed to guide use and minimize risks in this population. Our primary aim was to examine the potential interactive effect of regular cannabis use on chronic pain and sleep. A total of 339 nights (2273.43 ​h) of in-home sleep electroencephalogram (EEG) recordings were collected from 60 adults (50 ​% male; 32 ​% chronic pain; 47 ​% cannabis use; *M*_age_ ​= ​25.25; *SE* ​= ​1.05) over seven consecutive nights per participant. A mixed-model repeated-measures ANCOVA tested the main effects and interactions of chronic pain and regular cannabis use on total sleep time (TST), total slow-wave sleep (SWS%), total rapid-eye-movement (REM%), sleep onset latency (SOL), and number of awakenings. There was a significant main effect of cannabis use on SWS, TST, SOL, and REM. There was a significant main effect of chronic pain on TST. Significant interactions emerged between cannabis use and chronic pain on SWS and REM. These findings may reflect a dysregulated sleep response in individuals using cannabis to manage chronic pain, highlighting the need to consider both beneficial and detrimental effects of cannabis on specific sleep stages.

## Introduction

The most reported reasons for medicinal cannabis use are for pain relief and improvements in sleep [[1], [2], [3]], with some studies reporting more than half (i.e., 64 ​%) do so for pain [4], while others report up to 80 ​% use it for sleep [5,6]. The endocannabinoid system (ECS) plays a regulatory role in both pain and sleep [[7], [8], [9]]. Tetrahydrocannabinol (THC), the primary psychoactive ingredient in cannabis, binds with CB1 receptors in pain processing areas that lead to the reduction of nociceptive signaling [10,11], anti-inflammatory effects, and modulation of emotional response to pain [12]. In terms of sleep, THC activates CB1 receptors in brain regions like the hypothalamus, amygdala, and brainstem, which are involved in sleep regulation. Acute cannabis use has been associated with improvements in various sleep metrics including total sleep time (TST), sleep onset latency (SOL), slow-wave sleep (SWS), and number of sleep disruptions [5,13]. However, prolonged use (i.e, near daily for three months) potentially worsens sleep by reducing TST and SWS and increasing SOL [13]. Despite the role of ECS in both sleep and pain regulation [[7], [8], [9]], few studies have examined the combined effect of cannabis use and pain on sleep.

Existing studies, in general, demonstrate sleep improvements following cannabis therapy in those with chronic pain. For example, THC nasal spray administered to patients with neuropathic pain resulted in improvements in subjective sleep measures [14], which is concordant with the effects of synthetic THC in patients with fibromyalgia when compared to an antidepressant [15]. In another clinical trial, smoked cannabis reduced pain intensity and improved subjective sleep quality in patients with neuropathic pain [16]. Observational study findings suggest that among adults with chronic pain, only those who use cannabis have fewer night-time awakenings [3]. Interestingly, there also appears to be a dose effect such that more frequent use of cannabis increase wakefulness and sleep onset latency that could be related to potential cannabis tolerance [17]. Taken together, existing studies suggest that cannabis is associated with improvements in subjective measures of sleep, although its therapeutic effect may decline over time. To date, however, how these subjective effects relate to objective measures of sleep (i.e., sleep architecture) have yet to be examined.

Our review of the literature suggests that cannabis may provide a dual reinforcing effect in those with chronic pain by alleviating pain and improving sleep. However, given that improvements in sleep may diminish with tolerance related to more frequent or prolonged use [18,19], there is a risk of escalating use to maintain cannabis’s therapeutic effects. The goal of this study was to examine the potential interactive relationship between regular cannabis use, pain, and sleep. Previous research has shown that both chronic pain and cannabis use independently exert negative effects on sleep parameters, including slow-wave sleep (SWS) and rapid eye movement (REM) sleep [20], [21], [22], [23]. Building on these findings, we hypothesized an interaction between long-term cannabis use and chronic pain, such that individuals with chronic pain who regularly use cannabis would exhibit reduced SWS and REM sleep compared to those who do not use cannabis, regardless of pain status.

## Methods

The Institutional Review Board of the University of Texas at Dallas approved this study.

### Participants

Sixty-two participants were recruited from the general community within the Dallas metro area via flyers and social media (i.e., Facebook, Twitter, and Reddit). The inclusion criteria were: (a) aged 18–45 years, (b) proficiency in English, and (c) ability to provide informed consent. The exclusion criteria were: (a) history of brain injury or neurological diagnosis (e.g., stroke, epilepsy, MS), (b) history of psychosis, (c) currently taking psychotropic medications, (d) pregnancy, (e) history of sleep disorder, (f) shift-work (work hours between 10 p.m. and 6 a.m.), and (g) use of illicit substance other than cannabis.

### Study procedures and measures

The study consisted of an initial lab visit and seven days of in-home data collection. The initial lab visit included collection of informed consent and completion of self-report questionnaires. Age and gender were collected via demographics questionnaire. The Substance Use History Questionnaire (SUHQ) was used to assess frequency and duration of cannabis use and other substances. Regular cannabis use was defined as using at least three times a week for the past three months [24,25]. Chronic pain was defined based on ICD-11 (2021) definition of persistent pain for at least three months [26] as assessed using the following questions “Have you experienced persistent pain for the past 3 months or longer (i.e., chronic pain)?” and “What is the name of your pain condition. If unknown, please describe the pain you experience”.

EEG recordings were collected using the DREEM 3 headband [27]. The DREEM headband contains dry electrodes positioned at frontal (F7, F8, Fp2) and occipital (O1, O2) sites in addition to sensors to monitor heart rate, respiration, and movement. DREEM’s proprietary algorithms segmented sleep into the standard stages: wake, N1, N2, N3, and REM in addition to total sleep time, sleep efficiency, sleep onset latency, and the duration of each sleep stage. DREEM’s accuracy in sleep staging has been validated against polysomnography [27].

### Data processing and statistical analyses

We recorded 434 nights of sleep EEG. Of these sleep recordings, 95 nights of data were excluded due to poor data quality defined as a quality score of <70 ​% based on DREEM’s algorithm. As a result, two participants were excluded entirely from the analyses, resulting in a final sample of 60 participants with 339 nights (2273.43 ​h) of usable EEG sleep recordings. Among these participants, 16 reported nociceptive pain (10 of whom used cannabis), and 3 with cannabis use reported non-nociceptive pain (see Table 1 for sample characteristics).

**Table 1:** Sample characteristics.

Total (N)	60
Gender (M/F)	30/30
Age (M/SE)	25.25/1.05
Chronic pain	32 ​%
Regular cannabis use	47 ​%
Cannabis use years (M/SE)	3.1/0.88
Cannabis use days per week (M/SE)	3.41/0.66

Study sample characteristics of pain, gender, and cannabis use. Regular cannabis use was defined as using at least three times a week for the past three months.

Repeated measures analyses were performed using Linear Mixed Models (LMMs) in SPSS v.29 to take into account the unbalanced number of sleep recordings from each participant. The models included cannabis use (yes/no) and presence of pain (yes/no) as fixed effects, and subject ID as a random intercept to account for repeated measures nested within individuals. The dependent variables were TST, SOL, SWS, REM, and number of awakenings. We included gender and age as covariates of no interest (ANCOVA structure). Models were estimated using restricted maximum likelihood (RML) with a compound symmetry covariance structure, which provides less biased variance estimates and assumes equal correlations among repeated measures, yielding stable estimates in unbalanced within-subject data. As opposed to mean differences in sleep metrics, results from the RML models provide estimates of the fixed effects that are interpreted with the unstandardized coefficient “β” that indicate the magnitude and direction of the effect [28]. Effects were only deemed significant if zero was not included within the 95 ​% confidence interval and *p* ​< ​.05.

Considering longer durations of cannabis use diminish changes to sleep architecture [5,13] and cause worse sleep in those with chronic pain [17], Pearson correlations were computed to test for the effects of years of using cannabis on sleep metrics. Data reduction was performed by averaging the sleep metrics for those reporting regular cannabis use.

**Table 2:** Type III fixed effects and interactions for chronic pain and cannabis use on TST.

	*dfn*	*dfd*	*F*	*p*	β	*SE*	95 ​% CI	
							Lower bound	Upper bound
(Intercept)	1	36.54	480.34	<.001	338.85	25.26	288.49	389.21
**Main effects**								
Chronic pain	1	44.63	16.84	**<.001∗**	27.84	13.19	**1.32**	**54.36**
Cannabis use	1	45.95	6.52	**.01∗**	−39.21	16.75	**−73.01**	**−5.42**
**Interaction**								
Chronic pain x	1	44.45	1.70	.20	−26.20	20.12	−66.73	14.33
Cannabis use								
**Covariates**								
Sex	1	46.40	2.00	.16	12.92	9.13	−5.47	31.30
Age	1	51.19	2.68	.11	−16.70	10.20	−37.17	3.77

Results from the Linear Mixed Model that tested the main effects of chronic pain and cannabis use as well as the interaction of the two on total sleep time (TST). Significant effects are indicated with the *p* value and 95 ​% CI bolded. Chronic pain contributed to 28 ​min more TST, regular cannabis use contributed to ∼39 ​min less TST, and there was no interaction of cannabis use and chronic pain on TST. F ​= ​ratio of explained to unexplained variance for each predictor, dfn ​= ​the number of model parameters, dfd ​= ​estimated residual variance, β ​= ​unstandardized regression coefficient, SE ​= ​standard error, and 95 ​% CI ​= ​the confidence interval that provides the plausible range for the population effect.

## Results

The following results are based on the fixed factors and interactions between cannabis and chronic pain tested in the models. All main effects and interactions on TST, SOL, SWS, REM, and awakenings are reported in Table 2, Table 3, Table 4, Table 5, Table 6.

**Table 3:** Type III fixed effects and interactions for chronic pain and cannabis use on SOL.

	*dfn*	*dfd*	*F*	*p*	β	*SE*	95%CI	
							Lower bound	Upper bound
(Intercept)	1	64.15	.09	.76	−7.48	5.74	−18.89	3.93
**Main effects**								
Chronic pain	1	50.36	2.84	.10	−1.82	2.81	−7.47	3.83
Cannabis use	1	50.06	13.31	**<.001∗**	−10.34	3.67	**−2.96**	**−17.72**
**Interaction**								
Chronic pain x	1	48.89	.72	.40	−3.60	4.25	−12.15	4.94
Cannabis use								
**Covariates**								
Sex	1	50.48	.06	.82	−.44	1.88	−4.23	3.34
Age	1	50.53	.07	.79	.65	2.41	−4.20	5.50

Results from the Linear Mixed Model that tested the main effects of chronic pain and cannabis use as well as the interaction of the two on sleep onset latency (SOL). Significant effects are indicated with the *p* value and 95 ​% CI bolded. Regular cannabis use was associated with falling asleep ∼10 ​min faster, whereas chronic pain and the interaction of chronic pain and cannabis use were not significant predictors of sleep onset latency. F ​= ​ratio of explained to unexplained variance for each predictor, dfn ​= ​the number of model parameters, dfd ​= ​estimated residual variance, β ​= ​unstandardized regression coefficient, SE ​= ​standard error, and 95 ​% CI ​= ​the confidence interval that provides the plausible range for the population effect.

**Table 4:** Type III fixed effects and interactions for chronic pain and cannabis use on SWS.

	*dfn*	*dfd*	*F*	*p*	β	*SE*	95 ​% CI	
							Lower bound	Upper bound
(Intercept)	1	61.01	399.86	<.001	25.66	2.07	21.55	29.77
**Main effects**								
Chronic pain	1	37.71	17.25	**<.001∗**	−.92	1.13	−1.36	3.21
Cannabis use	1	39.16	7.33	**<.01∗**	5.08	1.44	**2.16**	**8**
**Interaction**								
Chronic pain x	1	36.02	9.85	**<.001∗**	5.35	1.70	**1.89**	**8.81**
Cannabis use								
**Covariates**								
Sex	1	36.73	4.35	**.04**	1.65	.79	**.05**	**3.26**
Age	1	44.88	.09	.77	−.29	.99	−2.29	1.71

Results from the Linear Mixed Model that tested the main effects of chronic pain and cannabis use as well as the interaction of the two on total sleep time slow-wave sleep (SWS). Significant effects are indicated with the *p* value and 95 ​% CI bolded. Chronic pain was associated with reduced SWS; however, the value of zero fell within the 95 ​% confidence interval, suggesting an unreliable effect. Regular cannabis use contributed to ∼5 ​% greater SWS, and a significant interaction showed that regular cannabis use and chronic pain contributed to greater amounts of SWS. F ​= ​ratio of explained to unexplained variance for each predictor, dfn ​= ​the number of model parameters, dfd ​= ​estimated residual variance, β ​= ​unstandardized regression coefficient, SE ​= ​standard error, and 95 ​% CI ​= ​the confidence interval that provides the plausible range for the population effect.

**Table 5:** Type III fixed effects and interactions for chronic pain and cannabis use on REM.

	*dfn*	*dfd*	*F*	*p*	β	*SE*	95 ​% CI	
							Lower bound	Upper bound
(Intercept)	1	89.89	256.03	<.001	19.57	2.40	14.82	24.33
**Main effects**								
Chronic pain	1	50.41	11.14	**<.001∗**	1.87	1.36	−.87	4.60
Cannabis use	1	53.96	8.01	**<.01∗**	−8.50	1.83	**−12.18**	**−4.83**
**Interaction**								
Chronic pain x	1	49.04	25.19	**<.001∗**	−10.73	2.14	**−15.03**	**−6.43**
Cannabis use								
**Covariates**								
Sex	1	52.33	2.12	.15	−1.38	.95	−3.28	.52
Age	1	53.06	.17	.68	.46	1.13	−1.80	2.73

Results from the Linear Mixed Model that tested the main effects of chronic pain and cannabis use as well as the interaction of the two on rapid eye movement (REM) sleep. Significant effects are indicated with the *p* value and 95 ​% CI bolded. Chronic pain contributed to a slight increase in REM sleep; however, the value of zero fell within the 95 ​% confidence interval, suggesting an unreliable effect. Regular cannabis use decreased REM by ∼8 ​%, and a significant interaction indicated that the effect of chronic pain and regular cannabis use reduced REM sleep by 10.73 ​%. F ​= ​ratio of explained to unexplained variance for each predictor, dfn ​= ​the number of model parameters, dfd ​= ​estimated residual variance, β ​= ​unstandardized regression coefficient, SE ​= ​standard error, and 95 ​% CI ​= ​the confidence interval that provides the plausible range for the population effect.

**Table 6:** Type III fixed effects and interactions for chronic pain and cannabis use on awakenings.

	*dfn*	*dfd*	*F*	*p*	β	*SE*	95 ​% CI	
							Lower bound	Upper bound
(Intercept)	1	68.27	140.09	<.001	20.08	2.38	15.36	24.80
**Main effects**								
Chronic pain	1	45.60	.60	.44	2.31	1.33	−4.98	.36
Cannabis use	1	47.80	1.68	.20	.08	1.82	−3.75	3.59
**Interaction**								
Chronic pain x	1	45.74	2.00	.16	3.00	2.12	−1.27	7.27
Cannabis use								
**Covariates**								
Sex	1	46.56	.18	.67	.38	.90	−1.43	2.20
Age	1	52.09	.02	.89	−.16	1.16	−2.49	2.16

Results from the Linear Mixed Model that tested the main effects of chronic pain and cannabis use as well as the interaction of the two on awakenings. No significant effects were indicated. F ​= ​ratio of explained to unexplained variance for each predictor, dfn ​= ​the number of model parameters, dfd ​= ​estimated residual variance, β ​= ​unstandardized regression coefficient, SE ​= ​standard error, and 95 ​% CI ​= ​the confidence interval that provides the plausible range for the population effect.

**Table 7:** Correlations between sleep metrics and years of regular cannabis use and pain.

Sleep Metrics	Years of Regular Use	Pain
SOL	.09	.05
TST	.27	−.1
SWS	**−.33∗**	.07
REM	−.07	.01
Awakenings	.08	−.1

Pearson’s one-tailed correlations between sleep metrics and years of regular cannabis use and pain. SOL ​= ​sleep onset latency, TST ​= ​total sleep time, SWS ​= ​slow-wave sleep, REM ​= ​rapid eye movement (∗*p* ​< ​.05).

### Main effects of cannabis on sleep metrics

The significant main effects of regular cannabis included: [1] lower TST (*β* ​= ​−39.21, 95 ​%; CI [−73.01, −5.42]), [2] decreased SOL (*β* ​= ​−10.34, 95 ​%; CI [−2.96, −17.72]), [3] increased SWS (*β* ​= ​5.08, 95%CI [2.16, 8]), and [4] less REM sleep (*β* ​= ​−8.5, 95%CI [−12.18,-4.83]). There was no significant effect of cannabis on awakenings (*β* ​= ​0.08, 95%CI [−3.75, 3.59]).

### Main effects of chronic pain on sleep metrics

There was a significant main effect of pain on TST such that chronic pain was associated with greater TST (*β* ​= ​27.84, 95%CI [1.32, 54.36]). No significant main effect of chronic pain was found on SOL (*β* ​= ​−1.82, 95%CI [−7.47, 3.83]), SWS (*β* ​= ​−0.92, 95%CI [−1.36, 3.21]), REM (*β* ​= ​1.87, 95%CI [−0.87, 4.6]), or awakenings (*β* ​= ​2.31, 95%CI [−4.98, 0.36]).

### Interactions of cannabis and chronic pain on sleep metrics

**Fig. 1:**
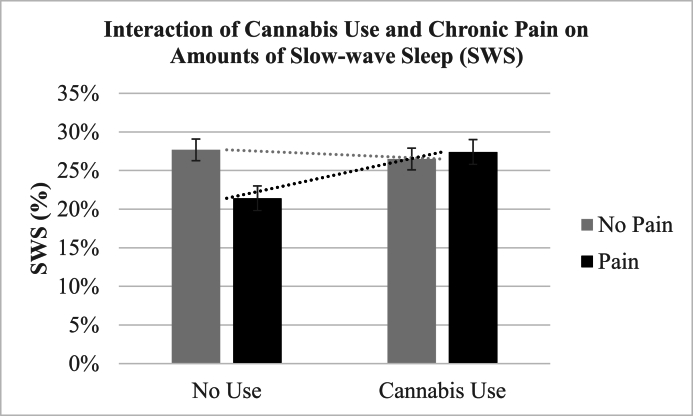
The interaction of regular cannabis use and chronic pain on the percentage of slow-wave sleep (SWS). The mean (SE) values were 27.41% (.02) for chronic pain and cannabis use, 26.49% (.01) for no pain and cannabis use, 27.68% (.01) for no pain and no cannabis use, and 21.41% (.02) for chronic pain and no cannabis use.

**Fig. 2:**
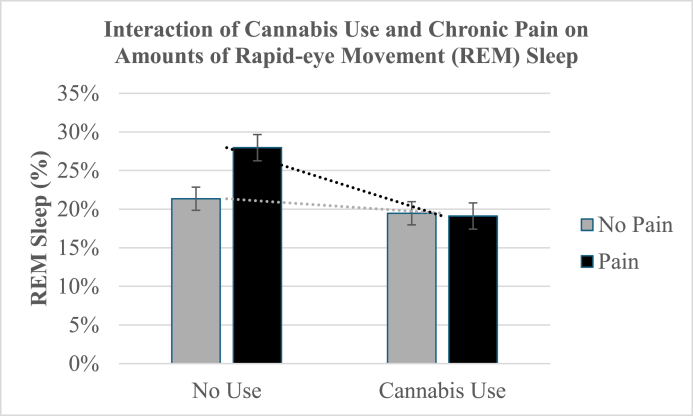
The interaction of regular cannabis use and chronic pain on the percentage of rapid-eye movement (REM) sleep. The mean (SE) values were 19.11% (.02) for chronic pain and cannabis use, 19.47% (.02) for no pain and cannabis use, 21.35% (.02) for no pain and no cannabis use, and 27.96% (.02) for chronic pain and no cannabis use.

There was a significant interaction between cannabis use and pain on: [1] SWS (*β* ​= ​5.35, 95%CI [1.89, 8.81]) such that cannabis use and chronic pain led to greater SWS (see Fig. 1); and [2] REM sleep (*β* ​= ​−10.73, 95%CI [−15.03, −6.43]) such that chronic pain and cannabis use both decreased amounts of REM sleep (see Fig. 2). No significant interactions were found between cannabis use and pain on TST (*β* ​= ​−26.2, 95%CI [−66.73, 14.33]), SOL (*β* ​= ​−3.6, 95%CI [−12.15, 4.94]), or awakenings (*β* ​= ​2.12, 95%CI [−1.27, 7.27]).

### Correlations between duration of cannabis use and sleep metrics

Results from the one-tailed Pearson correlations revealed a significant negative association between years of regular cannabis use and slow-wave sleep (SWS), such that more years of use was associated with reduced SWS (*p* ​= ​.04). There were no significant correlations between years of regular cannabis use and TST (*p* ​= ​.12), SOL (*p* ​= ​.32), awakenings (*p* ​= ​.38), or REM sleep (*p* ​= ​.36) See Table 7.

### Post-hoc analyses

We conducted linear mixed regression analyses to examine potential effects of type of pain on our outcomes. Our findings showed no significant effect of pain type (i.e., nociceptive vs. non-nociceptive pain types) on TST (*β* ​= ​−1.71, 95%CI [−87.71, 84.29]), SOL (*β* ​= ​−8.84, 95%CI [−25.19, 7.51]), SWS (*β* ​= ​−6.37, 95%CI [−13.31, 0.56]), REM sleep (*β* ​= ​−2.78, 95%CI [−12.43, 6.88]), or awakenings (*β* ​= ​4.93, 95%CI [−2.88, 12.73]).

## Discussion

The interactive effect of cannabis use and chronic pain on sleep remains unclear despite the acknowledged bi-directional relationship between pain and sleep (i.e., poor sleep increases pain sensitivity, and more pain worsens sleep) and the regulatory role of the endocannabinoid system on both. Our findings indicated that the combination of regular cannabis use and chronic pain was associated with increased SWS and decreased REM, suggesting that the effects of cannabis use on SWS and REM differ depending on the presence of chronic pain.

The interactive effect of cannabis and pain on sleep reflects the complex overlapping roles of the ECS in regulating pain, sleep, and their shared neurobiological pathways [29]. Specifically, THC interacts with CB1 receptors that are densely located in brain areas involved in both nociception and sleep regulation, such as the hypothalamus, amygdala, thalamus, and brainstem [8]. Studies suggest that THC attenuates pain signaling and inhibits GABA through the activation of CB1 receptors and indirectly by increasing anandamide (AEA) and 2-arachidonoylglycerol (2-AG) that complete retrograde synaptic signaling of CB1 receptors [30,31]. This increase of AEA and 2-AG from THC also promotes sleep [[30], [31], [32], [33]]. However, in individuals with chronic pain, the ECS is dysregulated, which could cause exogenous cannabinoids like THC to alter sleep architecture differently than in those without pain. Given that immune cell activation is reduced during SWS [34], increasing the amount of SWS may therefore provide better pain outcomes [35]. This suggests a negative feedback loop between sleep architecture and pain, such that pain increases sleep disruptions, decreases SWS, and triggers immune cell activation, which leads to increased inflammation and experienced pain [36,37]. Thus, the direct pain-alleviating qualities of THC [5,31,38] and indirect pain alleviation via increased SWS may benefit the regulation of pain.

The complex interaction between cannabis use and chronic pain in shaping SWS and REM suggests a potential tradeoff whereby increased SWS might be at the cost of impairing REM. SWS is critical for physical restoration and immune function [34], so this increase may reflect a compensatory mechanism to recover from the physiological strain of chronic pain, enhanced by cannabis. On the other hand, a marked reduction in REM sleep could indicate a disruption in cognitive or emotional restorative processes, which may have long-term implications for mental health and pain perception [39,40]. For example, reduced REM sleep impaired the integration of newly acquired non-declarative, procedural, and emotional memories [41] and compromises emotional regulatory mechanisms that contribute to heightened emotional reactivity and difficulty in coping with stressors [42]. fMRI findings show that reduced REM sleep increases amygdala activation and medial prefrontal cortex control during emotional tasks [39], which is similar to the functional connectivity dysregulation reported in individuals with chronic pain at rest [43].

Although our results of increased SWS and reduced REM sleep in those reporting chronic pain and regular cannabis use is consistent with findings of acute effects of cannabis on sleep [44], our correlational analyses revealed that greater years of regular cannabis use was associated with decreased SWS across all participants. This latter finding is consistent with evidence of tolerance and receptor downregulation that is observed from long-term cannabis use [20,45], suggesting that while cannabis may initially enhance SWS, these benefits diminish with chronic exposure. This finding provides further evidence for the biphasic effect of cannabis on sleep, where acute cannabis use promotes sleep through direct ECS modulation, while chronic use leads to tolerance, receptor downregulation, and disrupted sleep architecture. The paradoxical finding of reduced SWS with greater duration of cannabis use across all participants (i.e., those with pain and without pain) with observations of increased SWS in those with chronic pain may indicate that cannabis continues to provide some benefit for sleep through pain relief, even as tolerance develops to its direct effects on the ECS. By contrast, in individuals without chronic pain, cannabis effects on sleep are more direct, allowing the biphasic pattern of initial benefit (increased SWS with short-term use) followed by tolerance-related disruption (reduced SWS with long-term use) to emerge more clearly. This suggests that pain-related sleep disturbances may mask cannabis-related changes in sleep architecture. Our findings indicating no effect of pain type on our outcomes support previous reports of similar sleep disturbances across different pain diagnoses [46].

### Limitations and conclusions

Interpretation of these findings is limited by the study’s cross-sectional design. A longitudinal approach is needed to understand the potential parabolic function of cannabis use in populations with chronic pain. This knowledge could inform clinical monitoring of patients using cannabis therapeutically.

To conclude, these findings suggest a complex interaction between cannabis use and chronic pain in shaping sleep architecture, particularly in SWS and REM sleep. Our findings suggest that the interaction between cannabis and pain leads to a potential “trade-off” such that the beneficial sleep process is enhanced (SWS) while another critical function served by REM sleep may be compromised. Cannabis use in people with chronic pain may promote more deep, physically restorative sleep (SWS), but at the functional cost of reduced REM sleep, which could impair emotional and cognitive health over time. This trade-off could paradoxically worsen the long-term experience of pain, mood, or stress, despite short-term sleep improvements.

## Author Contributions

Tracy Brown: Conceptualization, Methodology, Data collection and curation, Analyses, Writing – original draft. Francesca Filbey: Conceptualization, Methodology, Data analysis consultation, Supervision, Writing – review & editing.

## Funding

This study was funded by the Bert Moore Endowed Chair.

## Declaration of competing interest

The authors declare that they have no known competing financial interests or personal relationships that could have appeared to influence the work reported in this paper.

## References

[1] Orhurhu V., Urits I., Olusunmade M., Olayinka A., Salisu Orhurhu M., Uwandu C. (2020 Aug 6). Cannabis use in hospitalized patients with chronic pain. Adv Ther.

[2] Lu Y., Anderson H.D. (2017). Cannabinoid signaling in health and disease. Can J Physiol Pharmacol.

[3] Sznitman S.R., Bretteville-Jensen A.L. (2015 Dec 14). Public opinion and medical cannabis policies: examining the role of underlying beliefs and national medical cannabis policies. Harm Reduct J.

[4] Kosiba J.D., Maisto S.A., Ditre J.W. (2019 Jul). Patient-reported use of medical cannabis for pain, anxiety, and depression symptoms: systematic review and meta-analysis. Soc Sci Med.

[5] Cranford J.A., Arnedt J.T., Conroy D.A., Bohnert K.M., Bourque C., Blow F.C. (2017 Nov). Prevalence and correlates of sleep-related problems in adults receiving medical cannabis for chronic pain. Drug Alcohol Depend.

[6] Bachhuber M., Arnsten J.H., Wurm G. (2019 Nov). Use of cannabis to relieve pain and promote sleep by customers at an adult use dispensary. J Psychoact Drugs.

[7] Haney M. (2022 Jan). Cannabis use and the endocannabinoid system: a clinical perspective. Am J Psychiatr.

[8] Murillo-Rodríguez E. (2008 Aug). The role of the CB1 receptor in the regulation of sleep. Prog Neuropsychopharmacol Biol Psychiatr.

[9] Vaughn L.K., Denning G., Stuhr K.L., de Wit H., Hill M.N., Hillard C.J. (2010 Jun 19). Endocannabinoid signalling: has it got rhythm?. Br J Pharmacol.

[10] Lynch M.E., Campbell F. (2011 Nov). Cannabinoids for treatment of chronic non-cancer pain; a systematic review of randomized trials. Br J Clin Pharmacol.

[11] Schley M., Legler A., Skopp G., Schmelz M., Konrad C., Rukwied R. (2006 Jul 31). Delta-9-THC based monotherapy in fibromyalgia patients on experimentally induced pain, axon reflex flare, and pain relief. Curr Med Res Opin.

[12] Romero-Sandoval E.A., Kolano A.L., Alvarado-Vázquez P.A. (2017 Oct 5). Cannabis and cannabinoids for chronic pain. Curr Rheumatol Rep.

[13] Kaul M., Zee P.C., Sahni A.S. (2021 Jan). Effects of cannabinoids on sleep and their therapeutic potential for sleep disorders. Neurotherapeutics.

[14] Rog D.J., Nurmikko T.J., Friede T., Young C.A. (2005 Sep 27). Randomized, controlled trial of cannabis-based medicine in central pain in multiple sclerosis. Neurology.

[15] Ware M.A., Fitzcharles M.A., Joseph L., Shir Y. (2010 Feb). The effects of nabilone on sleep in fibromyalgia: results of a randomized controlled trial. Anesth Analg.

[16] Ware M.A., Wang T., Shapiro S., Robinson A., Ducruet T., Huynh T. (2010 Oct 5). Smoked cannabis for chronic neuropathic pain: a randomized controlled trial. Can Med Assoc J.

[17] Sznitman S.R., Vulfsons S., Meiri D., Weinstein G. (2020 Dec). Medical cannabis and insomnia in older adults with chronic pain: a cross-sectional study. BMJ Support Palliat Care.

[18] Hasin D.S., Shmulewitz D., Cerdá M., Keyes K.M., Olfson M., Sarvet A.L. (2020 Jul 1). U.S. adults with pain, a group increasingly vulnerable to nonmedical cannabis use and cannabis use disorder: 2001–2002 and 2012–2013. Am J Psychiatr.

[19] Ramaekers J.G., Mason N.L., Theunissen E.L. (2020 Jul). Blunted highs: pharmacodynamic and behavioral models of cannabis tolerance. Eur Neuropsychopharmacol.

[20] Bolla K.I., Lesage S.R., Gamaldo C.E., Neubauer D.N., Funderburk F.R., Cadet J.L. (2008). Sleep disturbance in heavy marijuana users. Sleep.

[21] Older S.A., Battafarano D.F., Danning C.L., Ward J.A., Grady E.P., Derman S. (1998). The effects of delta wave sleep interruption on pain thresholds and fibromyalgia-like symptoms in healthy subjects; Correlations with insulin- like growth factor I. J Rheumatol.

[22] Onen S.H., Alloui A., Gross A., Eschallier A., Dubray C. (2001). The effects of total sleep deprivation, selective sleep interruption and sleep recovery on pain tolerance thresholds in healthy subjects. J Sleep Res.

[23] Cousens K., DiMascio A. (1973). (?)?9 THC as an hypnotic. Psychopharmacologia.

[24] Desrosiers N.A., Himes S.K., Scheidweiler K.B., Concheiro-Guisan M., Gorelick D.A., Huestis M.A. (2014 Apr 1). Phase I and II cannabinoid disposition in blood and plasma of occasional and frequent smokers following controlled smoked cannabis. Clin Chem.

[25] van der Pol P., Liebregts N., de Graaf R., Korf D.J., van den Brink W., van Laar M. (2013 Dec). Predicting the transition from frequent cannabis use to cannabis dependence: a three-year prospective study. Drug Alcohol Depend.

[26] Treede R.D., Rief W., Barke A., Aziz Q., Bennett M.I., Benoliel R. (2015). A classification of chronic pain for ICD-11. Pain.

[27] Arnal P.J., Thorey V., Debellemaniere E., Ballard M.E., Bou Hernandez A., Guillot A. (2020 Nov 12). The dreem headband compared to polysomnography for electroencephalographic signal acquisition and sleep staging. Sleep.

[28] Muth C., Bales K.L., Hinde K., Maninger N., Mendoza S.P., Ferrer E. (2016 Feb 21). Alternative models for small samples in psychological research. Educ Psychol Meas.

[29] Guindon J., Hohmann A. (2009 Dec 1). The endocannabinoid system and pain. CNS Neurol Disord: Drug Targets.

[30] Maccarrone M., Finazzi-Agró A. (2003 Sep 22). The endocannabinoid system, anandamide and the regulation of mammalian cell apoptosis. Cell Death Differ.

[31] Hill K.P., Palastro M.D., Johnson B., Ditre J.W. (2017 Jan). Cannabis and pain: a clinical review. Cannabis Cannabinoid Res.

[32] Murillo-Rodriguez E., Blanco-Centurion C., Sanchez C., Daniele P., Shiromani P.J. (2003 Dec). Anandamide enhances extracellular levels of adenosine and induces sleep: an in vivo microdialysis study. Sleep.

[33] Chester L.A., Englund A., Chesney E., Oliver D., Wilson J., Sovi S. (2024 Feb 1). Effects of cannabidiol and Delta-9-Tetrahydrocannabinol on plasma endocannabinoid levels in healthy volunteers: a randomized double-blind four-arm crossover study. Cannabis Cannabinoid Res.

[34] Ranjbaran Z., Keefer L., Stepanski E., Farhadi A., Keshavarzian A. (2007 Feb). The relevance of sleep abnormalities to chronic inflammatory conditions. Inflamm Res.

[35] Irwin M.R., Olmstead R., Bjurstrom M.F., Finan P.H., Smith M.T. (2023 May). Sleep disruption and activation of cellular inflammation mediate heightened pain sensitivity: a randomized clinical trial. Pain.

[36] Smith M.T., Haythornthwaite J.A. (2004). How do sleep disturbance and chronic pain inter-relate? Insights from the longitudinal and cognitive-behavioral clinical trials literature. Sleep Med Rev.

[37] Young K., Del Fabbro E., Giurgiutiu D.V., Healy W.J. (2024 Dec). Sleep disturbances in chronic pain. ATS Sch.

[38] Cuttler C., LaFrance E.M., Craft R.M. (2022 Feb 1). A large-scale naturalistic examination of the acute effects of cannabis on pain. Cannabis Cannabinoid Res.

[39] van der Helm E., Yao J., Dutt S., Rao V., Saletin J.M., Walker M.P. (2011 Dec). REM sleep depotentiates amygdala activity to previous emotional experiences. Curr Biol.

[40] Boyce R., Williams S., Adamantidis A. (2017 Jun). REM sleep and memory. Curr Opin Neurobiol.

[41] Ackermann S., Rasch B. (2014 Feb 7). Differential effects of Non-REM and REM sleep on memory consolidation?. Curr Neurol Neurosci Rep.

[42] Goldstein A.N., Walker M.P. (2014 Mar 28). The role of sleep in emotional brain function. Annu Rev Clin Psychol.

[43] Mao C.P., Yang H.J., Yang Q.X., Sun H.H., Zhang G.R., Zhang Q.J. (2022 Feb). Altered amygdala-prefrontal connectivity in chronic nonspecific low back pain: resting-state fMRI and dynamic causal modelling study. Neuroscience.

[44] Babson K.A., Sottile J., Morabito D. (2017). Cannabis, cannabinoids, and sleep: a review of the literature. Curr Psychiatry Rep.

[45] Garcia A.N., Salloum I.M. (2015 Oct). Polysomnographic sleep disturbances in nicotine, caffeine, alcohol, cocaine, opioid, and cannabis use: a focused review. Am J Addict.

[46] Finan P.H., Goodin B.R., Smith M.T. (2013 Dec). The association of sleep and pain: an update and a path forward. J Pain.

